# Tribological Properties of Cu-MoS_2_-WS_2_-Ag-CNT Sintered Composite Materials

**DOI:** 10.3390/ma15238424

**Published:** 2022-11-26

**Authors:** Adam Piasecki, Mateusz Kotkowiak, Maciej Tulinski, Robert Čep

**Affiliations:** 1 Institute of Materials Science and Engineering, Faculty of Materials Engineering and Technical Physics, Poznan University of Technology, Piotrowo 3, 60-965 Poznan, Poland; 2 Department of Machining, Assembly and Engineering Metrology, Faculty of Mechanical Engineering, VSB-Technical University of Ostrava, 17. Listopadu 2172/15, 708-00 Ostrava, Czech Republic

**Keywords:** self-lubricating sinters, silver, carbon nanotubes, tungsten disulfide, molybdenum disulfide, wear resistance, COF

## Abstract

In this work, in order to produce Cu-MoS_2_-WS_2_-Ag-CNT self-lubricating materials, powder metallurgy was used. Several different compositions containing single solid lubricant MoS_2_, WS_2_, Ag and CNTs as well as multi-component lubricants in the copper matrix were prepared. Friction and wear tests were carried out using the pin-on-disc method at room temperature. Light microscopy (LM), scanning electron microscopy (SEM), energy dispersive spectroscopy (EDS) and X-ray diffraction (XRD) were used to characterize the wear mechanism of sintered materials. The tribofilm on the worn surfaces of sintered materials and counter-specimens was observed. The influence of single solid lubricants and the synergistic interaction of two, three or four solid lubricants on tribological properties of sintered composite materials were determined.

## 1. Introduction

Frictional wear is the main cause of wear of machine parts and tools. It is estimated to be responsible for 80% of their breakdowns. Approximately 30% of energy is used to overcome frictional resistance. Annual economic losses due to frictional wear account for almost 2% of national GDP. Therefore, studies on friction and the search for new materials, including lubricating oils, greases and solid lubricants, are a topical issue of important social and economic benefits. With developing industry and modern production technologies, requirements for wear resistance in demanding operating conditions, such as high speeds, high loads, high vacuum, radiation and operating temperature range, are increased. This means a need to search for new materials, and modification of their surfaces by appropriate treatments [1,2,3,4,5]. Nevertheless, the issue of lubrication is an indispensable element of cooperation of these materials in friction nodes. Lubrication oils, plastic greases and solid lubricants can be distinguished. Currently, there is a tendency to replace lubricating oils and plastic greases with solid lubricants [6,7,8,9,10]. Oils and plastic greases can pose a serious problem in some applications, for example, at high temperatures, when they may evaporate. In addition, they cannot be used for a long time in a high vacuum greater than 10^−1^ Pa. Lubricating oils pollute the environment during their production, and practically at all stages of their use: in transport to users, in long-term storage, during operation and at their disposal. Oils, aromatic and unsaturated hydrocarbons and heterogeneous compounds (containing sulfur, nitrogen and oxygen) are particularly harmful to humans. The solution may be to use solid lubricants. Solid lubrication technology was first used in military industry, and then in advanced space, aviation and electronics industries. This solved many problems that could not be solved with oil lubrication. Currently, an intense search for self-lubricating materials and surface layers that contain solid lubricants embedded in their matrix is underway [11,12,13,14,15]. They can be produced using: powder metallurgy (resulting in the formation of self-lubricating materials), laser alloying (resulting in the formation of technological superficial layers) or thermal spraying as well as PVD and CVD techniques (resulting in the formation of coatings) [11,16,17,18,19,20,21,22,23]. The disadvantages of self-lubricating coatings are limited service life, difficulties in refilling, oxidation, aging-related degradation and poor adhesion. To avoid the disadvantages of both lubricating oils or greases and self-lubricating coatings, producing self-lubricating materials containing solid lubricants in a metal matrix seems to be promising [24]. Powder metallurgy is of particular interest as it results in a homogeneous composite in which particles of solid lubricant will be evenly distributed, which may in turn allow a formation of tribofilm between mating parts during operation. Solid lubricants can be divided into four groups: soft metals, metal compounds, organic materials and inorganic materials [25,26]. In addition, they may be divided depending on application temperature: the first group from −200 °C to room temperature, second group from room temperature to 500 °C and third group working over 500 °C [27].

In this study, eight sintered composite materials containing solid lubricants such as WS_2_, MoS_2_, CNTs, Ag and copper, which constituted the sinter matrix, were examined. Copper and silver have high thermal conductivity, which ensures heat dissipation from the friction area of mating parts. Moreover, they have excellent electrical properties. Lubricating properties are related to their low shear strength and easy intercrystalline slip. During the friction, they form on the surfaces a film that reduces coefficient of friction (COF) and, as a consequence, diminishes the wear. The higher the purity of soft metal, the lower the shear strength and the easier the slip in the soft metal. Tungsten disulfide (WS_2_) has the lowest dry friction coefficient of approximately 0.03. It has higher thermal stability than that of MoS_2_, and has a layered structure. It can be used at high temperature, under high pressure, in high vacuum and under heavy load. It is chemically inert, non-toxic and non-corrosive to metal [7,26,28,29,30]. Molybdenum disulfide (MoS_2_) also has a layered structure. It is the most commonly used solid lubricant. It is even incorporated in lubricating oils and greases [26,31,32]. Moreover, there is a considerable interest in modifying oils and greases with carbon nanotubes (CNTs). Introducing them into lubricating oils reduces friction and wear of mating parts [33,34]. Due to their excellent physical properties, low density, high aspect ratio and tribological properties, carbon nanotubes are also used as reinforcement in composite materials [24,35,36,37,38]. The effect of single solid lubricant additions has been widely researched. However, taking into account the different conditions of the investigation, the topic still remains relevant. There are also studies of the influence of two or more solid lubricants on the tribological properties of the produced materials. The studies by Kong et al. [39] have shown that the introduction of titanium into the composite of Ni20Cr + 15 wt. % WS_2_ resulted in a reduction of the friction coefficient from 0.53 to 0.37. The use of nano-Cu and h-BN as solid lubricants in nickel-based coating also had a positive effect on the COF value over a wide temperature range [13]. Lu et al. [30] have produced Cu/RGO/WS_2_ composites, which were characterized by a reduced COF, which was approximately 0.2. They have found that in the case of the addition of only WS_2_ in the amount of 40%, the friction coefficient was higher and was approx. 0.3–0.4. In the work [40], the friction and wear characteristics of Ni-Al-Ag-MoS_2_, Ni-Al-Ag-MoS_2_—5 wt.% hBN and Ni-Al-Ag-MoS_2_—10 wt.% hBN coatings were tested. The friction coefficients obtained at room temperature were in the range of 0.45–0.65. Ni_3_Al-WS_2_-Ag-hBN composites have been tested by Shi et al. [41]. The friction coefficients at room temperature ranged from 0.48 to 0.61.

An interesting group of materials that can contain solid lubricants in their structure are high-entropy alloys (HEAs) [15,42,43]. Zhang et al. [15] produced CoCrFeNi HEA composites containing silver and BaF_2_/CaF_2_ eutectic as solid lubricants using the spark plasma sintering method (SPS). Ag was assigned the main role in reducing the coefficient of friction of the composite at temperatures ranging from RT to about 400 °C. The good lubricity of the composite from 400 to 800 °C was mainly attributed to the combined lubricating effect of Ag, BaF_2_/CaF_2_ eutectic and various metal oxides that formed on the surface of the composite at high temperatures.

The works cited as well as the works of other authors prove that the search for new self-lubricating materials is still valid. The objective of this study was to produce sintered composite materials containing solid lubricants by powder metallurgy and to investigate their tribological properties. The frictional wear tests were carried out under dry friction conditions at room temperature. Light microscopy (LM), scanning electron microscopy (SEM), energy dispersive spectroscopy (EDS) and X-ray diffraction (XRD) were used for the research.

## 2. Materials and Methods

### 2.1. Sinter Preparation

**Figure 1:**
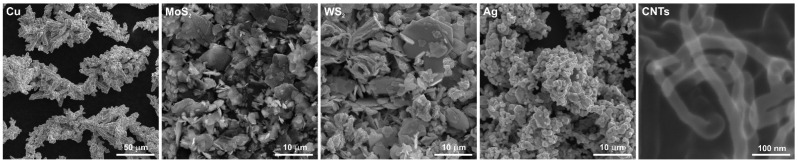
The powders used to make the sinters.

In the first step, powder mixes were tumbled for 1 h. The chemical composition of powder mixes used in order to produce the sinters is shown in Table 1. Figure 1 shows morphologies of the powders used. Copper powder had a dendritic shape. The shapes of the MoS_2_ and WS_2_ powders were different, as they are characterized by a flake structure. Silver powder was in the form of agglomerates and had a spherical shape. Carbon nanotubes, just as silver powder, were in the form of agglomerates. The diameter of the carbon nanotubes was approximately 30 nm. In the second stage, powder metallurgy compacts were made of powders with a copper matrix and solid lubricants. Powder mixture pressing was carried out at a pressure of 1.17 GPa. In the next stage, sintering was carried out at 800 °C for 3 h in a tube furnace under argon atmosphere. Sinter cooling was carried out in the furnace at a 250 °C/h rate. The produced sinters had a diameter of 4 mm and a height of 5 mm. To ensure clarity of this article, only the percentage is used, without “wt.”.

### 2.2. Wear Tests

Measurement of tribological properties of the sinters at room temperature was carried out on a tribometer (T-21, Lukasiewicz Research Network—IST, Poland) in a pin-on-disc arrangement. Commercially available Inconel^®^625 with a diameter of 25.4 mm and a height of 4 mm was used as counter-specimens. Frictional tests in dry friction conditions were carried out under a load of 5 N for 1 h at a rotational speed of 120 min^−1^. Each of the wear tests was repeated three times. During the tests, friction forces were recorded for friction pairs: sintered material (pin)–Inconel^®^625 (disc). The friction coefficients were determined using the equation:

$$\mu =\frac{F_{f}}{F_{l}}$$

where: *μ*—friction coefficient; *F_f_*—friction force [N]; *F_l_*—load applied [N].

The results of wear tests were presented in the form of graphs showing the friction coefficients vs. the time of friction as well as the mass change of specimens and counter-specimens for all the composite materials produced.

### 2.3. Tests of Worn Surfaces and of Worn Debris

The worn surfaces of sintered materials and counter-specimens as well as wear products were observed after the tests using SEM (Mira 3, Tescan, the Czech Republic) equipped with EDS (Ultim Max 65, Oxford Instruments, Abingdon, UK). In order to limit the excitation zone, an accelerating voltage of 12 kV was used. Quantitative tests of chemical composition of the surface of sintered materials and wear products were carried out. The element concentrations were also distributed in the form of EDS maps by means of a color scale. In the color scale, the colors corresponded to the concentration of the selected element. The intensive color pixels indicated the highest concentration of the element, and the darker areas corresponded to the concentration near to zero. Numerical values on these maps correspond to the number of counts. The phase composition tests were carried out on sinter surfaces after the frictional tests using an X-ray diffractometer (Empyrean, PANalytical, the Netherlands) equipped with a copper lamp (X-ray wavelength λ = 1.54060 Å) and an X’Celerator detector. XRD patterns were recorded at room temperature, in the angular range 25–100° 2theta, in steps of 0.017° 2theta. The phase analysis of the obtained diffractograms was performed in the HighScore program equipped with the PDF-4 database.

## 3. Results and Discussion

**Figure 2:**
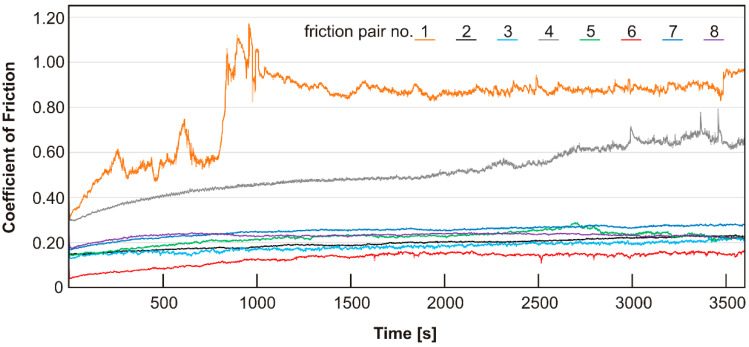
Friction coefficient vs. time of friction self-lubricating composite mating with Inconel^®^625 alloy at room temperature.

The results of the tests of friction coefficient as a function of time are shown in Figure 2. The highest coefficient of friction was observed for the specimen with a copper matrix with the addition of silver (sinter no. 1) and it was about 0.89, similar to pure copper. In the work [44], the authors obtained a friction coefficient of 0.7–0.75 for pure copper in a friction pair with WC-Co, while for the Cu-5% Ag alloy it was in the range of 0.6–0.8. Such high coefficients of friction of pure metals or metal alloys are caused by plastic deformation, caused by sliding and subsequent changes in the microstructure beneath the worn surface [45,46]. During sliding, unevenness shear occurs, which affects the roughness of the surface. Repeated sliding on a rough surface generates wear particles. This leads to mechanical alloying and welding of these particles to the worn surface, creating the nanostructured tribolayers [47,48,49,50]. The delamination of brittle tribolayers during a subsequent slip leads to a further increase in surface roughness and a high COF. As for the remaining specimens with a single additive, a gradual increase in COFs is observed and some stabilization takes place at the final stage of the study. In the case of the Cu + 20% MoS_2_ specimen (sinter no. 2), the friction coefficient changes from 0.15 to 0.23, and for the Cu + 20% WS_2_ specimen (sinter no. 3) it was in the range of 0.13–0.22. The friction pair with the specimen containing MoS_2_ was characterized by a smoother COF curve than that of the specimen containing WS_2_. Furlan et al. [51] have extensively reviewed self-lubricating composites containing only MoS_2_ ranging from 1–90 wt. % as well as additional ingredients. Depending on the matrix and the test conditions, the friction coefficients ranged from 0.05 to 0.55. In the work [52], it was shown that MoS_2_ magnetron-sputtered coatings were characterized by friction coefficients at the level of 0.03–0.04. In the case of using the friction pair consisting of a Cu + 20% WS_2_ specimen and 100Cr6 steel as a counter-specimen, the friction coefficient obtained by Freschi et al. [28] was higher than that in this study, and was approximately 0.35. Xiao et al. [53] have investigated the effect of the amount of WS_2_ on the lubricating properties of Cu-WS_2_ composites. The composite with 10 wt.% WS_2_ was characterized by a 71% reduction in COF, and the addition of 40% WS_2_ reduced the wear rate by 96%.

For the specimen with carbon nanotubes (sinter no. 4), the coefficient of friction was in the range of 0.3–0.65. The beneficial effect of carbon nanotubes could result from a twofold impact, namely, they may introduce rolling motion or they may be degraded and smeared on the worn surface [24,36]. The resulting tribofilm on the contact surface between the mating metallic surfaces prevented direct metal-to-metal contact and reduced the adhesion between the contact surfaces [54]. The increase in the friction coefficient to about 0.6 after approximately 2500 s and its increased fluctuations after approximately 3000 s probably resulted from the partial removal of carbon nanotubes from the contact zone and the breaking of the tribofilm [24,36].

In the case of sinter 5 containing 5% MoS_2_ and 5% WS_2_, the friction coefficient stabilized at 0.23. Modification of sinter 5 by an addition of 2% silver (sinter no. 6) resulted in a reduction of the friction coefficient to a value of about 0.15. The addition of carbon nanotubes to the sinter containing 5% MoS_2_, 5% WS_2_ and 2% Ag (sinter no. 7) allowed us to obtain a friction coefficient of 0.27 in the final stage of the tests. Fluctuations in the coefficient of friction were negligible for this specimen and oscillated within the range of 0.02 for each test range. Modification of sinter 5 by the addition of carbon nanotubes allowed us to obtain a sinter characterized by a smoothed curve illustrating changes in the coefficient of friction and the shortest running-in period of approximately 500 s. This specimen was not characterized by the lowest coefficient of friction among the tested specimens. COF value was at the level of about 0.24, and in the final stage of the test it diminished to 0.22. However, based on the course of friction coefficient changes, it should be concluded that lubricating properties of this sinter were very good. The advantageous effect of carbon nanotubes in composites containing sulfides (sinter no. 7) as well as sulfides and silver (sinter no. 8) could be related to their difficult removal from the friction track during the test and the formation of a multi-component tribofilm.

**Figure 3:**
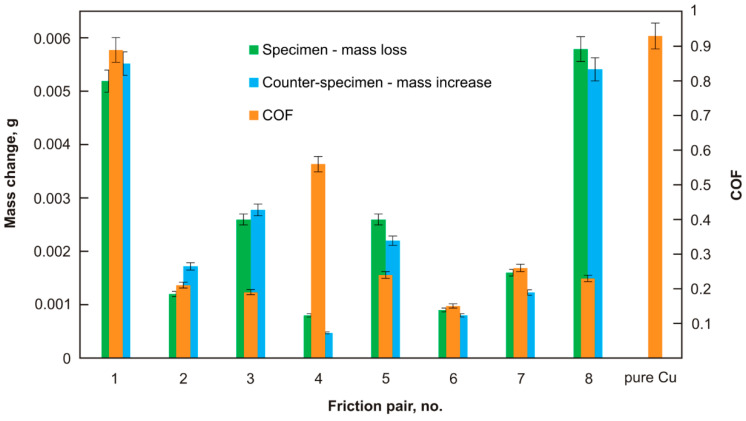
The average friction coefficients and mass changes.

The specimens and counter-specimens were weighed prior to and following the frictional tests in order to determine the changes in their mass. Weight loss was observed for all the tested specimens, while weight gain was found for all the counter-specimens (Figure 3). The changes in the mass of each friction pair were comparable, i.e., a loss in mass of the specimen was similar to an increase in mass of the counter-specimen. The lowest weight losses were found for sinters Cu + 2% CNTs (sinter no. 4) and Cu + 5% MoS_2_ + 5% WS_2_ + 2% Ag (sinter no. 6), and were, respectively: 0.8 mg and 0.9 mg. The weight gains of the counter-specimens were 0.5 mg and 0.8 mg, respectively. It should be noted that the Cu + 2% CNTs sinter was characterized by one of the highest friction coefficients measured. The highest weight loss of the specimens (5.2 mg and 5.5 mg) and the increase in the weight of counter-specimens (5.8 mg and 5.5 mg) were recorded for the Cu + 10% Ag specimen (sinter no. 1) and sinter no. 8, i.e., Cu + 5% MoS_2_ + 5% WS_2_ + 2% CNTs, respectively. 

**Figure 4:**
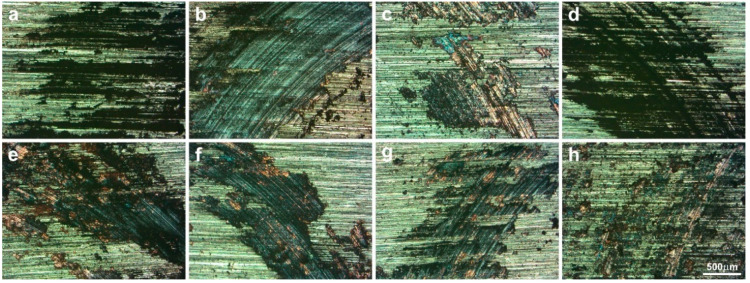
Worn surfaces of the counter-specimens tested at RT (LM); friction pair no. 1 (**a**), 2 (**b**), 3 (**c**), 4 (**d**), 5 (**e**), 6 (**f**), 7 (**g**) and 8 (**h**).

Figure 4 shows friction tracks on counter-specimens, which prior to the tests were subjected to grinding. All the tested counter-specimens showed horizontal grinding traces in friction tracks. As these marks were not fully destroyed during the operation of the friction pair, it proved very good tribological properties of the sinters tested in this study. Copper was visible in the friction tracks, so during the frictional wear test of the friction pairs, some matrix material was also removed and rubbed into the friction track. This proved adhesive wear. Under the influence of friction force, the worn surface was subjected to shear stresses along the sliding direction and was torn off the surface and then spread over the counter-specimen surface. The most continuous tribofilm on the surface was observed for the counter-specimen cooperating with sinter no. 2—Cu + 20% MoS_2_. The friction pair with the highest coefficient of friction, i.e., the pair with a specimen with only silver addition (sinter no. 1), was characterized by filling the cavities on the counter specimen after grinding the worn debris. Unlike the first sinter, in other friction tracks, clear traces of solid lubricants being smeared and micro-scratches on the resulting tribofilms were not visible apart from filling the grooves after the grinding process. In the work [55], the laser-textured surfaces entrapped CNTs and provided long-term lubrication. In the present study, the grooves on the surface of the counter-specimens after grinding played a similar role.

**Figure 5:**
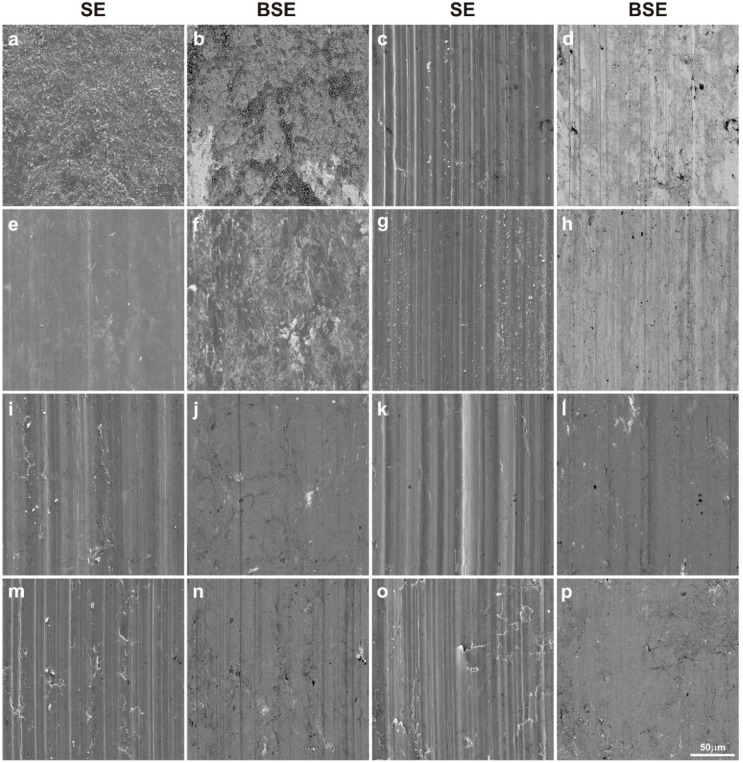
Worn surfaces of the sinters tested at RT (SEM); friction pair no. 1 (**a**,**b**), 2 (**c**,**d**), 3 (**e**,**f**), 4 (**g**,**h**), 5 (**i**,**j**), 6 (**k**,**l**), 7 (**m**,**n**) and 8 (**o**,**p**).

Figure 5 shows SEM images of sinter surfaces after the wear tests. They were taken in contrast of secondary electrons (SEs) and backscattered electrons (BSEs). The purpose of the BSE contrast test was to detect possible chemical composition differences on sinter surfaces. Darker areas contained lighter elements, and lighter ones—heavier ones. On the worn surface of the Cu + 10% Ag sinter, a large portion of worn debris in the form of loose particles was observed, that could be responsible for the high coefficient of friction. In BSE contrast, besides dark areas, there were also lighter ones. It proved a certain spreading of wear products and their oxidation in darker areas. The other specimens were characterized by different worn surfaces, where traces of micro-cutting or micro-ploughing could be seen to a greater or lesser extent. For the Cu + 20% MoS_2_ specimen (sinter no. 2), the tribofilm in the form of darker areas was visible in the BSE contrast. As can be seen, the tribofilm did not completely cover the specimen surface. Moreover, its thickness was very small. 

**Figure 6:**
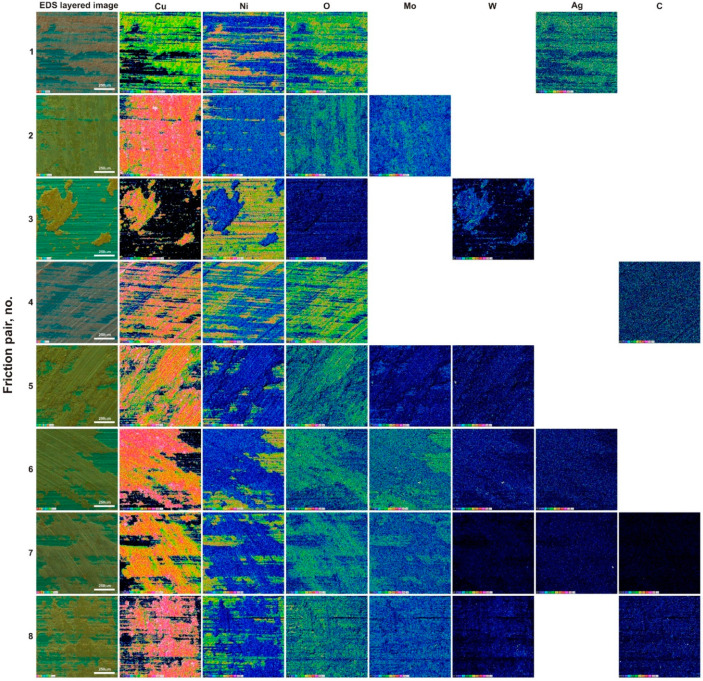
Worn surfaces of the counter-specimens (Inconel^®^625 alloy) tested at RT (EDS maps).

**Figure 7:**
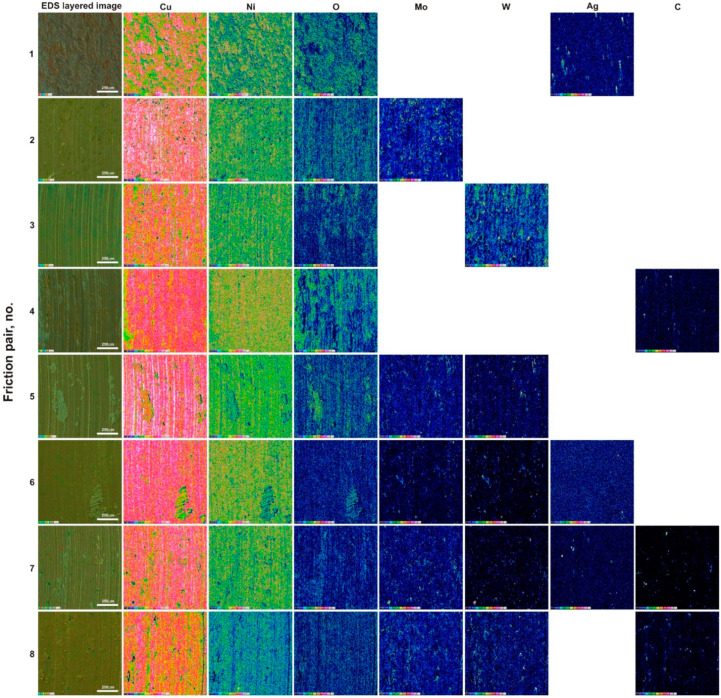
Worn surfaces of the specimens (sintered composite materials) tested at RT (EDS maps).

The BSE image largely reflected the observations in secondary electron contrast (SE). The surface of the specimen containing only WS_2_ (sinter no. 3) as an additive was very smooth, and only a few micro-scratches were visible. The BSE surface image of this sinter showed the distribution of WS_2_ (light areas). Lubricant spreading was clearly visible without any obvious directivity. This proves the excellent lubricating properties of WS_2_. Sinter no. 4 with carbon nanotubes had the worn surface similarly to the MoS_2_ specimen (sinter no. 2). The micro-scratches were characterized by a smaller depth and were thinner. In addition, a higher content of very fine worn debris was observed. In the BSE image, a tribofilm of uniform thickness was visible. The gray color variation could correspond to the tribofilm thickness variation. So, in brighter areas the tribofilm could have a lower thickness. As for the specimen containing two types of sulfides (sinter no. 5), apart from very fine micro-scratches, a small worn debris content could be noticed, and in the BSE contrast, a uniform gray color could be observed. The addition of 2% Ag (sinter no. 6) resulted in the appearance of traces of micro-ploughing. In BSE contrast, a continuous tribofilm was observed and traces of friction wear were poorly visible. In the specimen containing all lubricant additives (sinter no. 7), micro-scratches, a few loose particles and some cracks in the resulting tribofilm were visible. What could probably have happened in this case was that during the test a strengthening, and then adhesive detachment and the formation of a new tribofilm layer, occurred. The micro-scratches visible in the BSE image indicated a relatively low thickness of the tribofilm. However, it should be noted that there was practically one shade of gray on the specimen. A similar situation was observed in the SE image for the specimen containing 5% MoS_2_, 5% WS_2_ and 2% CNTs (sinter no. 8), with a greater number of micro-scratches. Tribofilm delamination was also visible. The BSE image showed a homogeneous gray image with no clear scratches. Thus, in this case a tribofilm of uniform thickness was obtained. Figure 6 and Figure 7 show the EDS maps on the worn surfaces of counter-specimens and sinters, respectively. EDS maps were made in a 12-point color scale. The areas with the highest content of the analyzed element were visible as white, then in colors showing a lower content, successively light pink, pink, red, orange, yellow, light and dark green, purple, dark blue and black. The black area indicated the absence of the element. The values entered in the successive rectangles of different colors show the number of counts for a given element. As could be seen, copper was visible in each surface analyzed. Thus, during the wear by friction, the sinters were sheared, and then the copper matrix and lubricants, which were released from the worn debris and the sinters themselves, were smeared. This was evidenced by the appearance on maps of those elements that were part of lubricants in areas beside copper traces. Oxygen was found in friction tracks in all the counter-specimens, that proved oxidation wear. The lowest oxygen presence was found in the specimen Cu + 20% WS_2_ (sinter no. 3)—see map in dark colors. Oxygen distribution coincided with copper distribution. It means that copper was mainly oxidized. The highest oxygen content was found for specimens 1 and 4. Probably, the Ag and CNT particles contributed to an increased removal of tribofilm layers largely made up of copper as well as to its intense oxidation. Obviously, this could have an advantageous effect on tribological properties, because the copper oxide thus formed also became a lubricant. Nickel’s presence on sinter surfaces proved that, to some extent, abrasive or adhesive wear of counter-specimens occured throughout friction. The highest oxygen content was found on the surfaces of sinters 1, 2, 4 and 5. Specimens 6-8 were subjected to relatively low oxidation. The distribution of the elements’ concentrations on EDS maps, derived from solid lubricants on the entire analyzed surface of the sinters, proved the formation of a continuous tribofilm on them. 

**Figure 8:**
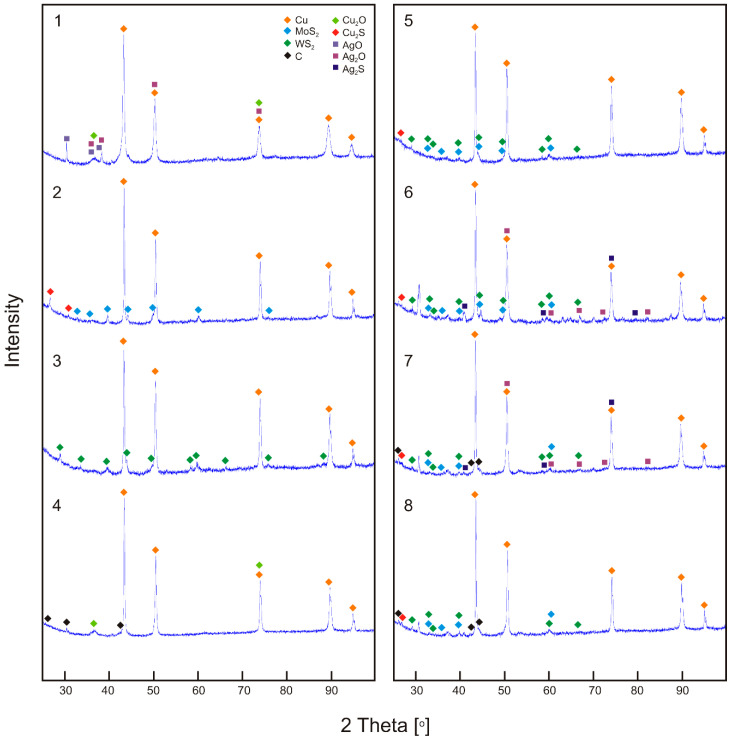
The XRD patterns of worn surface of sinters; friction pair no. 1–8.

The results of XRD phase composition analysis, performed on the sintered surfaces after the friction test, are shown in Figure 8. The worn surfaces tested consisted mainly of the components of the produced sinters. Pure copper was found in all the tested sinter surfaces. On the surface of Cu + 10% Ag (sinter no. 1) and Cu + 2% CNTs (sinter no. 4) specimens, the presence of copper oxide (Cu_2_O) was confirmed. Copper oxide was probably also present on the other worn surfaces of the sinters. However, due to the penetration depth of X-rays of the order of several dozens of µm combined with the very small thickness of the copper oxide, it could be invisible in XRD patterns for other sinters. A similar situation could be in the case of phases with nickel. EDS analysis showed the presence of Ni on the worn surfaces of the sinters, while in the XRD patterns no nickel phases, including nickel oxides, were found. Ag_2_O and AgO oxides were found on the surface of the sinters containing silver (1, 6 and 7), while silver sulfide (Ag_2_S) was found when the sinter contained sulfides besides silver (sinters 6 and 7). CuS_2_ was found on the worn surfaces for all the sinters containing MoS_2_. 

**Figure 9:**
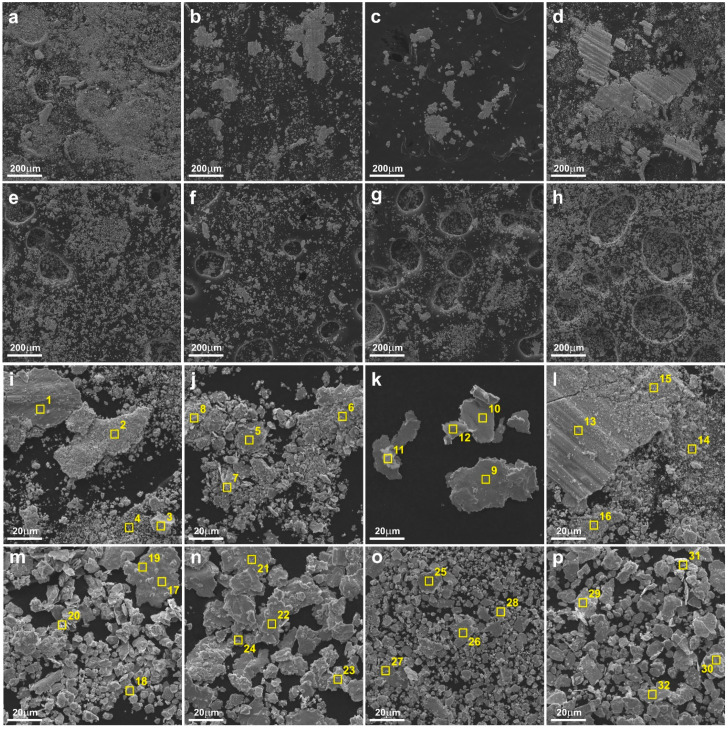
Worn debris with marked areas of EDS analysis (according to Table 2); friction pair no. 1 (**a**,**i**), 2 (**b**,**j**), 3 (**c**,**k**), 4 (**d**,**l**), 5 (**e**,**m**), 6 (**f**,**n**), 7 (**g**,**o**), 8 (**h**,**p**).

Figure 9 shows the worn debris, while Table 2 shows the results of testing their chemical composition using the EDS method. During the friction test between the components of the friction pairs, worn debris were formed, which were systematically removed from the friction track. However, some worn debris could remain on the friction track as a result of the difficulty in being removed beyond it. This was observed primarily in the case of friction pairs 1 and 4. The presence of worn debris in the friction track, especially of larger sizes and in larger amounts, increased the COF. Worn debris were collected from the friction node immediately post-friction wear test. The lowest amount of wear products was found in the friction pair composed of sinter 3, i.e., Cu + 20% WS_2_. The highest worn debris amount was observed in the friction pair composed of sinter containing copper and 2% CNTs (sinter no. 4). A significant amount of wear products was also found in the friction pair with Cu + 10% Ag (sinter no. 1). In addition to fine particles, a small amount of larger particles with a size of approximately 60 µm was detected. In a friction pair with sinter no. 2 (Cu + 20% MoS_2_), a smaller amount of wear products of various sizes was observed. Only fine worn debris was observed in the remaining friction pairs. As for the mating Inconel^®^625 with multi-component sinters, the lowest amount of worn debris was found in the Cu + 5% MoS_2_ + 5% WS_2_ + 2% Ag + 2% CNTs friction pair (sinter no. 7). Chemical composition tests confirmed the presence of oxygen in wear products in all the friction pairs. The largest oxygen content was observed in the friction pair with sinters 1 and 4. The presence of oxygen in worn debris indicated the oxidation wear in friction pair operation, that was consistent with the XRD tests performed on the worn surfaces of the sinters. In addition, in friction pairs 1 and 4 nickel was found in worn debris, obtaining 1.3-1.9 and 1.7-4.8 wt.%, respectively. This could prove that the Inconel^®^625 counter-specimens were more intensively subjected to abrasion. The lowest nickel content, within measurement error, was found in worn debris detected in friction pairs 3 and 5. In all wear products, all the elements included in the chemical composition of individual sinters were found.

## 4. Conclusions

In this work, sinters with a copper matrix containing solid lubricants such as Ag, MoS_2_, WS_2_ and CNTs were produced by powder metallurgy. On the basis of the research, the following conclusions were formulated:

- The lowest coefficient of friction (0.15) was observed for Cu + 5% MoS_2_ + 5% WS_2_ + 2% Ag sinter. The coefficient of friction for the sinter with silver addition was similar to that of pure copper and was 0.89. The friction coefficient for the sinter with carbon nanotubes was 0.56.
- The multi-component sinters with the addition of CNTs were characterized by smoothed curves of changes in the coefficient of friction vs. the time of friction.
- The lowest weight losses were found for Cu + 2% CNTs and Cu + 5% MoS_2_ + 5% WS_2_ + 2% Ag sinters.
- A mass increase was found for all the counter-specimens as a result of spreading sinter components, including the copper matrix, on their surfaces at a contact point of the friction pair.
- The Cu + 10% Ag sinter was characterized by the highest amount and greatest size of worn debris.
- The main wear mechanisms of the produced sinters were micro-cutting and micro-ploughing. In addition, adhesive and oxidation wear were observed.
- On the worn surfaces of sinters and counter-specimens, the presence of the tribofilm was observed. Primary traces of the grinding process in the friction tracks indicated a low wear of counter-specimens.

## Figures and Tables

**Table 1:** The chemical composition of powder mixes used in order to produce the sinters.

	Chemical Composition [wt. %]				
No.	Cu	MoS_2_	WS_2_	Ag	CNTs
1	bal.			10	
2	bal.	20			
3	bal.		20		
4	bal.				2
5	bal.	5	5		
6	bal.	5	5	2	
7	bal.	5	5	2	2
8	bal.	5	5		2

**Table 2:** The chemical composition of worn debris (wt.%).

Friction Pair No.	Point	Cu	Ni	O	Mo	W	Ag	S	C
1	1	78.7	1.9	12.7			6.7		
	2	79.1	1.7	12.4			6.8		
	3	79.9	1.3	12			6.7		
	4	81.5	1.5	10.4			6.6		
2	5	87.8	0.2	3.4	5.2			3.4	
	6	83.2	0.3	5	6.6			4.8	
	7	86.6	0	5.3	5.1			3	
	8	82.8	0.2	6.6	6.3			4.1	
3	9	85.1	0	1.3		10.1		3.5	
	10	78.3	0.1	1.4		15.8		4.5	
	11	79.2	0.1	3.3		12.8		4.7	
	12	77	0.1	1.6		16.5		4.8	
4	13	80.7	2.7	10.9					5.8
	14	76.8	1.7	10.1					11.4
	15	81.7	2.4	10.2					5.8
	16	80.5	4.8	8.9					5.9
5	17	94.8	0	1.7	1.1	1.4		1	
	18	87.2	0.3	3.9	2.3	3.7		2.8	
	19	72.6	0	1.7	0.3	20		5.4	
	20	85	0	7.9	1.9	2.9		2.2	
6	21	87	0.6	3.3	1.9	3.5	1.8	1.9	
	22	91.5	0.5	2.2	1.2	1.7	1.6	1.4	
	23	83.1	0	5.7	1.5	4.9	2.2	2.6	
	24	83.6	0.9	2.6	1.6	6.5	2	2.8	
7	25	75	0.7	2.1	2.8	5.5	1.7	3.7	8.7
	26	77	0.9	5.8	1.9	3.1	1.7	2.2	7.4
	27	75.2	0.6	3.1	1.6	5.5	1.5	2.4	9.9
	28	76.6	1.3	2.9	1.9	3.7	1.4	2	10.1
8	29	76	0.3	4.3	1.9	4.3		2.4	10.8
	30	73.2	0.4	3.1	3	4.4		2.9	13
	31	74.3	0.5	4.8	2.8	3.7		2.6	11.3
	32	73.9	0.1	3.3	2.3	8.4		2.6	9.4

## Data Availability

Not applicable.

## References

[1] Wu M., Pan L., Duan H., Wan C., Yang T., Gao M., Yu S. (2021). Study on Wear Resistance and Corrosion Resistance of HVOF Surface Coating Refabricate for Hydraulic Support Column. Coatings.

[2] Omrani E., Moghadam A.D., Kasar A.K., Rohatgi P., Menezes P.L. (2021). Tribological Performance of Graphite Nanoplatelets Reinforced Al and Al/Al_2_O_3_ Self-Lubricating Composites. Materials.

[3] Wang X., Tang B., Wang L., Wang D., Dong W., Li X. (2022). Microstructure, Microhardness and Tribological Properties of Bronze–Steel Bimetallic Composite Produced by Vacuum Diffusion Welding. Materials.

[4] Zhang R., Chen B., Liu F., Sun M., Zhang H., Wu C. (2022). Microstructure and Mechanical Properties of Composites Obtained by Spark Plasma Sintering of Ti_3_SiC_2_-15 vol.% Cu Mixtures. Materials.

[5] Piasecki A., Kotkowiak M., Makuch N., Kulka M. (2019). Wear behavior of self-lubricating boride layers produced on Inconel 600-alloy by laser alloying. Wear.

[6] Waqas M., Zahid R., Bhutta M.U., Khan Z.A., Saeed A. (2021). A Review of Friction Performance of Lubricants with Nano Additives. Materials.

[7] Hu N., Zhang X., Wang X., Wu N., Wang S. (2020). Study on Tribological Properties and Mechanisms of Different Morphology WS_2_ as Lubricant Additives. Materials.

[8] Kosarchuk V., Chausov M., Pylypenko A., Tverdomed V., Maruschak P., Vasylkiv V. (2022). Increasing Wear Resistance of Heavy-Loaded Friction Pairs by Nanoparticles in Conventional Lubricants: A Proof of Concept. Lubricants.

[9] Wang Y., Gao X., Lin J., Zhang P. (2022). Rheological and Frictional Properties of Lithium Complex Grease with Graphene Additives. Lubricants.

[10] Tóth Á.D., Szabó Á.I., Leskó M.Z., Rohde-Brandenburger J., Kuti R. (2022). Tribological Properties of the Nanoscale Spherical Y_2_O_3_ Particles as Lubricant Additives in Automotive Application. Lubricants.

[11] Piasecki A., Kulka M., Kotkowiak M. (2016). Wear resistance improvement of 100CrMnSi6-4 bearing steel by laser boriding using CaF_2_ self-lubricating addition. Tribol. Int..

[12] Tang H., Cao K., Wu Q., Li C., Yang X., Yan X. (2011). Synthesis and tribological properties of copper matrix solid self-lubricant composites reinforced with NbSe_2_ nanoparticles. Cryst. Res. Technol..

[13] Zhao Y., Feng K., Yao C., Nie P., Huang J., Li Z. (2019). Microstructure and tribological properties of laser cladded self-lubricating nickel-base composite coatings containing nano-Cu and h-BN solid lubricants. Surf. Coat. Technol..

[14] Elkady O.A.M., Abu-Oqail A., Ewais E.M.M., El-Sheikh M. (2015). Physico-mechanical and tribological properties of Cu/h-BN nanocomposites synthesized by PM route. J. Alloys Compd..

[15] Zhang A., Han J., Su B., Meng J. (2017). A novel CoCrFeNi high entropy alloy matrix self-lubricating composite. J. Alloys Compd..

[16] Piasecki A., Kotkowiak M., Kulka M. (2017). Self-lubricating surface layers produced using laser alloying of bearing steel. Wear.

[17] Kotkowiak M., Piasecki A., Kulka M. (2019). The influence of solid lubricant on tribological properties of sintered Ni–20% CaF_2_ composite material. Ceram. Int..

[18] Piasecki A., Kotkowiak M., Kulka M. (2017). The effect of CaF_2_ and BaF_2_ solid lubricants on wear resistance of laserborided 100CrMnSi6-4 bearing steel. Arch. Mater. Sci. Eng..

[19] Wen S., Dai C., Mao W., Ren Z., Wang X., Zhao Y., Han G. (2022). Microstructure and Wear Properties of HVAF Sprayed Cu-Zr-Al-Ag-Co Amorphous Coatings at Different Spray Temperatures. Coatings.

[20] Zhang R., Zhang H., Liu F. (2022). Microstructure and Tribological Properties of Spark-Plasma-Sintered Ti_3_SiC_2_-Pb-Ag Composites at Elevated Temperatures. Materials.

[21] Ye F., Lou Z., Wang Y., Liu W. (2022). Wear mechanism of Ag as solid lubricant for wide range temperature application in micro-beam plasma cladded Ni60 coatings. Tribol. Int..

[22] Wang D., Chen W., Sun Q., Wang L., Zhu S., Cheng J., Yang J. (2021). Tribological properties of Ni3Al-Ni3Nb-Ag self-lubricating alloys at a wide temperature range. Wear.

[23] Mi P., Ye F. (2018). Wear performance of the WC/Cu self-lubricating textured coating. Vacuum.

[24] Moghadam A.D., Omrani E., Menezes P.L., Rohatgi P.K. (2015). Mechanical and tribological properties of self-lubricating metal matrix nanocomposites reinforced by carbon nanotubes (CNTs) and graphene—A review. Compos. Part B Eng..

[25] Gong H., Yu C., Zhang L., Xie G., Guo D., Luo J. (2020). Intelligent lubricating materials: A review. Compos. Part B Eng..

[26] Kumar R., Hussainova I., Rahmani R., Antonov M. (2022). Solid Lubrication at High-Temperatures—A Review. Materials.

[27] Yang J.F., Jiang Y., Hardell J., Prakash B., Fanget Q.F. (2013). Influence of service temperature on tribological characteristics of self-lubricant coatings: A review. Front. Mater. Sci..

[28] Freschi M., Di Virgilio M., Haiko O., Mariani M., Andena L., Lecis N., Kömi J., Dotelli G. (2022). Investigation of second phase concentration effects on tribological and electrical properties of Cu–WS_2_ composites. Tribol. Int..

[29] Gao Q., Yan H., Qin Y., Zhang P., Guo J., Chen Z., Yu Z. (2019). Laser cladding Ti-Ni/TiN/TiW + TiS/WS_2_ self-lubricating wear resistant composite coating on Ti-6Al-4V alloy. Opt. Laser Technol..

[30] Lu D., Qian G., Feng Y., Zhao H., Zhou Z., Zhang X. (2020). Tribological Behaviors of Cu/RGO/WS_2_ Composites in Air and Vacuum Environments. Tribol. Trans..

[31] Tonge P., Roy A., Patel P., Beall C.J., Stoyanov P. (2022). Tribological Evaluation of Lead-Free MoS_2_-Based Solid Film Lubricants as Environmentally Friendly Replacements for Aerospace Applications. Lubricants.

[32] Amini M., Azadegan B., Akbarzadeh H., Gharaei R. (2022). Analysis of MoS_2_ and WS_2_ nano-layers role on the radiation resistance of various Cu/MS2/Cu and Cu/MS_2_@Cu@MS_2_/Cu nanocomposites. Materialia.

[33] Kałużny J., Kulczycki A., Dzięgielewski W., Piasecki A., Gapiński B., Mendak M., Runka T., Łukawski D., Stepanenko O., Merkisz J. (2020). The Indirect Tribological Role of Carbon Nanotubes Stimulating Zinc Dithiophosphate Anti-Wear Film Formation. Nanomaterials.

[34] Wu S., Tian S., Menezes P.L., Xiong G. (2020). Carbon solid lubricants: Role of different dimensions. Int. J. Adv. Manuf. Technol..

[35] Aristizabal K., Katzensteiner A., Bachmaier A., Mücklich F., Suarez S. (2017). Study of the structural defects on carbon nanotubes in metal matrix composites processed by severe plastic deformation. Carbon.

[36] Aristizabal K., Tayrac A., Katzensteiner A., Bachmaier A., Suarez S. (2019). Friction and Tribo-Chemical Behavior of SPD-Processed CNT-Reinforced Composites. Lubricants.

[37] Reinert L., Suárez S., Rosenkranz A. (2016). Tribo-Mechanisms of Carbon Nanotubes: Friction and Wear Behavior of CNT-Reinforced Nickel Matrix Composites and CNT-Coated Bulk Nickel. Lubricants.

[38] Bastwros M.M.H., Esawi A.M.K., Wifi A. (2013). Friction and wear behavior of Al–CNT composites. Wear.

[39] Kong X., Liu Y., Chen M., Zhang T., Wang Q., Wang F. (2022). Heterostructured NiCr matrix composites with high strength and wear resistance. J. Mater. Sci. Technol..

[40] Gautam R.K.S., Rao U.S., Tyagi R. (2019). High temperature tribological properties of Ni-based self-lubricating coatings deposited by atmospheric plasma spray. Surf. Coat. Technol..

[41] Shi X., Song S., Zhai W., Wang M., Xu Z., Yao J., Qamar Ud Din A., Zhang Q. (2014). Tribological behavior of Ni3Al matrix self-lubricating composites containing WS_2_, Ag and hBN tested from room temperature to 800 °C. Mater. Des..

[42] Cheng Z., Wang S., Wu G., Gao J., Yang X., Wu H. (2022). Tribological properties of high-entropy alloys: A review. Int. J. Miner. Metall. Mater..

[43] Ding H., Bao X., Jamili-Shirvan Z., Jin J., Deng L., Yao K., Gong P., Wang X. (2021). Enhancing strength-ductility synergy in an ex situ Zr-based metallic glass composite via nanocrystal formation within high-entropy alloy particles. Mater. Des..

[44] Chen X., Han Z. (2021). A low-to-high friction transition in gradient nano-grained Cu and Cu-Ag alloys. Friction.

[45] Rigney D.A. (2000). Transfer, mixing and associated chemical and mechanical processes during the sliding of ductile materials. Wear.

[46] Chen X., Han Z., Li X., Lu K. (2016). Lowering coefficient of friction in Cu alloys with stable gradient nanostructures. Sci. Adv..

[47] Kim H.J., Karthikeyan S., Rigney D. (2009). A simulation study of the mixing, atomic flow and velocity profiles of crystalline materials during sliding. Wear.

[48] Rigney D.A., Karthikeyan S. (2010). The Evolution of Tribomaterial During Sliding: A Brief Introduction. Tribol. Lett..

[49] Singh J.B., Wen J.G., Bellon P. (2008). Nanoscale characterization of the transfer layer formed during dry sliding of Cu–15wt.% Ni–8wt.% Sn bronze alloy. Acta Mater..

[50] Chen X., Han Z., Lu K. (2014). Wear mechanism transition dominated by subsurface recrystallization structure in Cu–Al alloys. Wear.

[51] Furlan K.P., de Mello J.D.B., Klein A.N. (2018). Self-lubricating composites containing MoS_2_: A review. Tribol. Int..

[52] Seynstahl A., Krauß S., Bitzek E., Meyer B., Merle B., Tremmel S. (2021). Microstructure, Mechanical Properties and Tribological Behavior of Magnetron-Sputtered MoS_2_ Solid Lubricant Coatings Deposited under Industrial Conditions. Coatings.

[53] Xiao J., Zhang W., Zhang C. (2018). Microstructure evolution and tribological performance of Cu-WS_2_ self-lubricating composites. Wear.

[54] Rajkumar K., Aravindan S. (2011). Tribological studies on microwave sintered copper–carbon nanotube composites. Wear.

[55] Reinert L., Lasserre F., Gachot C., Grützmacher P., MacLucas T., Souza N., Mücklich F., Suarez S. (2017). Long-lasting solid lubrication by CNT-coated patterned surfaces. Sci. Rep..

